# Exploring the Neural Pathways of Faith: A Review and Case Study on Hyperreligiosity in Epilepsy

**DOI:** 10.3390/neurosci7010004

**Published:** 2026-01-02

**Authors:** Guillermo José Bazarra Castro, Carlos Martínez Macho, Ricardo Mantecón Zorrilla, Enrique Barbero Pablos, Cristina V. Torres Díaz, Jose Antonio Fernández-Alén, Ricardo Gil Simoes

**Affiliations:** Department of Neurosurgery, Hospital Universitario de La Princesa, 28006 Madrid, Spain; guillebazarra@hotmail.com (G.J.B.C.);

**Keywords:** uncinate fasciculus, temporal lobe epilepsy, religious seizures, mystical seizures, ecstatic seizures, limbic network, frontotemporal connectivity, diffusion tensor imaging, tractography

## Abstract

Religious experiences represent a universal and timeless phenomenon that has accompanied humanity since its origins. In recent decades, neuroscience has explored the relationship between temporal lobe epilepsy (TLE) and hyperreligiosity phenomena, describing sudden convictions, states of ecstasy, and spiritual conversions associated with epileptic seizures. This article offers a narrative review of the literature on the relationship between epilepsy and religion, including its clinical manifestations (ictal, postictal, and interictal) and the main neurobiological models proposed to explain it, such as the limbic marker hypothesis and theory of mind (ToM). The possible role of the uncinate fasciculus as an integrative pathway between temporal and limbic regions is also explored, based on recent neuroimaging studies. Finally, we present an illustrative clinical case of a patient with meningioma and TLE associated with episodes of intense religious conviction, in whom a structural alteration of the right uncinate fasciculus was observed. This case reinforces the relevance of considering both neuronal networks and white matter tracts in the study of religious experiences, while underscoring the need for broader and more systematic studies to confirm these findings.

## 1. Introduction

The search for the meaning of existence and the belief in the transcendent constitute some of the most distinctive traits of human beings. Religion, as a manifestation of this search, has shaped the development of civilizations, permeating philosophy, art, and culture. In this context, a key question for contemporary neuroscience is how the brain generates the experience of the sacred.

We know that all conscious experience—from sensory perception to dreamlike states or religious convictions—is associated with the activation of specific brain networks [1]. Religious experiences are no exception, and numerous studies have investigated their neurobiological correlates.

One of the most striking phenomena described in neurology is hyperreligiosity associated with temporal lobe epilepsy (TLE). For decades, it has been observed that some patients with epilepsy, especially of temporal origin, experience sudden religious convictions, mystical visions, ecstatic states, and even radical conversions [2,3,4,5,6]. Examples described in the literature include brief but overwhelming convictions of divine presence or revelation, often accompanied by certainty of having received a message or mission [4]; mystical visions, such as the perception of luminous figures, angelic forms, or sacred symbols during focal impaired awareness seizures [3,7]; ecstatic states characterized by intense feelings of peace, unity, harmony, or “ultimate reality,” as documented in classical descriptions of ecstatic epilepsy [7,8,9]; and radical conversions, in which patients adopt new spiritual beliefs or markedly change their religious identity following a cluster of temporal seizures [4,5,6]. These examples illustrate the broad spectrum of religious-type experiences reported in temporal lobe epilepsy and help to frame the phenomena described in the present case.

The present work aims to:

1. Review the historical and clinical literature on the relationship between epilepsy and religion.
2. Describe the different forms of manifestation of hyperreligiosity in TLE.
3. Analyze the main neurobiological models proposed.
4. Present an illustrative clinical case, documenting a structural alteration of the uncinate fasciculus in a patient with episodes of religious conviction.

To support the narrative review presented in this article, we conducted a structured literature search using PubMed. The following keywords and combinations were applied: “temporal lobe epilepsy”, “hyperreligiosity”, “religious experiences”, “ecstatic seizures”, “uncinate fasciculus”, “frontotemporal connectivity”, and “limbic network”. The search was restricted to studies published in English or Spanish from 1990 to 2025. Additional relevant articles were identified through cross-referencing of bibliographies. Inclusion criteria comprised original clinical studies, neuroimaging research, case reports, and theoretical or review papers directly addressing epilepsy-related religious phenomena or the functional anatomy of the uncinate fasciculus. Exclusion criteria were non-scientific sources, articles without a neurological or neuroimaging focus, and publications not involving human participants. This approach ensured a comprehensive yet focused synthesis of the available evidence.

For the purposes of this article, the terms “religious experience” and “religiosity” are used in a phenomenological and neurocognitive sense, without reference to any specific religious tradition. We employed “religious” to denote experiences involving themes such as transcendence, divine presence, ultimate meaning, or spiritual revelation, regardless of the patient’s cultural or theological background. This operational definition allows us to describe the neural mechanisms associated with such experiences while avoiding assumptions linked to particular belief systems or denominational practices.

## 2. Epilepsy and Religion Throughout History

The association between epilepsy and religious experiences is not a modern finding but dates back to the earliest historical and cultural records of humanity. The interpretation of epileptic seizures has varied over the centuries, oscillating between supernatural conceptions and medical approaches, but in all cases, the extraordinary and often transcendent character of these manifestations has been acknowledged.

### 2.1. Antiquity: From the Divine to the Pathological

Historical interpretations of epilepsy as a supernatural or divine condition are well documented in classical medical and anthropological scholarship. Temkin’s comprehensive history of epilepsy [10] details how cultures from ancient Mesopotamia to Greece conceptualized seizures as manifestations of divine intervention, demonic possession, or sacred illness, supporting the notion that epilepsy oscillated between religious reverence and social marginalization. These sources provide historical confirmation for the coexistence of supernatural and proto-medical explanations during antiquity.

### 2.2. Middle Ages and Renaissance

The coexistence of mystical interpretations and proto-clinical observations during the medieval and Renaissance periods is well supported in historical analyses. Flanagan’s biography of Hildegard of Bingen [11] and the neurological reassessment by Brigo et al. [12] detail how visionary experiences were often interpreted within theological frameworks but may reflect migraine or epileptic auras. Furthermore, Schildkrout’s historical review of Joan of Arc [13] and the psychological studies of medieval mysticism by Kroll and Bachrach [14] illustrate how religious experiences, visions, and trance states were commonly embedded in spiritual discourse while still displaying features compatible with neurological phenomena. These works support the statement that interpretations in this period ranged from sanctity to suspicion of demonic influence.

### 2.3. Paradigmatic Cases in Religious History

- The Apostle Paul: His visions, recounted in the letters of the New Testament, such as the rapture to the “third heaven” (2 Corinthians 12:2–4), have been reinterpreted by modern neurology as possible psychic auras characteristic of temporal epilepsy [2]. This interpretation is further supported by Brorson and Brewer [15], who provide a detailed neurological reassessment of Paul’s visionary episodes, arguing that several features are compatible with psychic auras characteristic of temporal lobe epilepsy.
- Saint Teresa of Ávila: Her mystical ecstasies, described in detail in *The Book of Her Life*, display features compatible with “ecstatic epilepsy”: alterations of consciousness, sensations of levitation, and union with God [3]. A recent neurological reassessment by Huberfeld et al. [16] further explores the ecstatic episodes described by Teresa of Ávila, arguing that several phenomenological elements may align with temporal lobe epileptic activity while acknowledging the difficulty of distinguishing between epilepsy and genuine mystical ecstasy.
- Fyodor Dostoevsky: The Russian novelist suffered from temporal epilepsy and described in his works experiences of absolute fullness preceding seizures. Gastaut [7] described this as the clearest example of ecstatic epilepsy in literature.

### 2.4. Modern Interpretations

With the rise of neurology in the 19th and 20th centuries, the clinical view of epilepsy became consolidated. However, authors such as Jackson and Hughlings already noted the emotional and spiritual dimension of some temporal seizures, laying the foundations for the modern semiology of religious ictal experiences.

Contemporary studies, such as those by Devinsky and Lai [4] or Trimble [17], have documented cases of patients who, during seizures or in the interictal period, experience intense religious convictions, mystical visions, or sudden conversions. These modern clinical observations confirm what was intuitively perceived since antiquity: temporal lobe epilepsy can induce highly intense religious experiences, with implications not only medical but also cultural and spiritual. In this context, the term “cultural” refers to the shared symbols, narratives, and interpretative frameworks through which societies understand and classify extraordinary or transcendental experiences. The term “spiritual” denotes the subjective, experiential dimension of such events, including personal meaning, existential significance, and the sense of connection to a transcendent reality. Together, these elements shape how individuals interpret religious or mystical sensations arising from neurological phenomena.

## 3. Phenomenology of Hyperreligiosity in Temporal Lobe Epilepsy

Temporal lobe epilepsy (TLE) has long been considered the neurological model most closely linked to religious phenomena. Various authors have noted that patients with this form of epilepsy exhibit, more frequently than other groups, mystical-type experiences, sudden religious convictions, or even stable changes in their spiritual orientation [4,5,6].

Devinsky and Lai [4] proposed a classification of religious experiences in patients with focal epilepsy—especially temporal—into three broad categories: ictal, postictal, and interictal. These categories reflect not only the moment when they occur but also the duration, intensity, and life impact of the experiences.

### 3.1. Ictal Experiences

Ictal religious experiences occur during the epileptic episode, generally as part of a focal impaired awareness seizure. They may manifest as:

- Subjective sensations of divine presence: patients describe the certainty that a transcendent entity is next to them, without requiring vision or voice.
- Religious visions: appearance of images with spiritual content, such as angelic figures, saints, or sacred symbols.
- Mystical or emotional ecstasy: feelings of unity with the universe, absolute fullness, or indescribable peace.
- Auditory phenomena: voices transmitting religious messages or divine commands.

Their prevalence ranges between 0.4% and 3.1% of patients with focal epilepsy, being more frequent in those with right temporal foci [4]. Studies using systematic questionnaires have shown higher percentages than those obtained by spontaneous reports, suggesting that such experiences may be underreported.

Clinically, these experiences are brief, lasting seconds to minutes, but their emotional intensity is such that patients recall them as transcendent life experiences. Some state that those moments were “more real than reality itself.”

A classic example is that described by Gastaut regarding Dostoevsky: the writer experienced, just before his generalized seizures, a state of ecstasy with a sensation of universal harmony and mystical fullness, which he himself described in his literary works. This phenomenon has been considered the paradigm of “ecstatic epilepsy” [7].

### 3.2. Postictal Experiences

Postictal religious experiences appear in the period immediately following the seizure and are usually more prolonged than ictal ones. In this context, patients may present with:

- Religious delusions: beliefs of divine mission, messianic convictions, or persecutory ideas with spiritual content.
- Prolonged mystical states: sensations of enlightenment, certainty of having received revelations, or a compulsive need to preach.
- Thought disturbances: discourse centered on transcendental topics, with heavy emotional charge and poor critical capacity.

Their duration may range from several hours to days, sometimes associated with postictal psychosis. During these phases, patients often also present with behavioral disturbances, insomnia, and affective lability.

It is estimated that 1.3% of epileptic patients present with postictal religious phenomena, increasing to 2.2% in cases of TLE [4]. The temporal relationship between increased seizure frequency and religious conversion has been described on multiple occasions, suggesting a causal link.

A case documented by Koenig [10] described a patient who, after repeated seizures, developed convictions of a divine mission that persisted for days. This condition was interpreted as postictal psychosis with religious content.

### 3.3. Interictal Religiosity

Unlike ictal and postictal experiences, interictal religiosity represents a stable and persistent change in the patient’s personality and belief system. This phenomenon, classically described by Waxman and Geschwind [6], is part of an interictal behavioral syndrome associated with TLE, which includes the following:

- Hyperreligiosity: intense convictions, increased spiritual practice, sense of transcendent mission.
- Hypergraphia: compulsion to write, often on philosophical, moral, or religious topics.
- Moralizing and circumstantial discourse: tendency to elaborate lengthy ethical and spiritual reasoning, with difficulty in synthesizing.
- Decreased sexual interest: displacement of libido toward spiritual or abstract interests.

Bear and Fedio [5] found that patients with TLE, especially with right hemisphere involvement, showed a greater inclination toward religiosity compared with controls and other neurological groups. These changes may manifest as religious conversions, stronger commitment to faith communities, or even adoption of roles as preacher or spiritual guide.

Interictal religiosity is perhaps the most intriguing form of the phenomenon, since it implies a lasting transformation of the patient’s identity. In some cases, it persists even after seizure control, raising questions about the neurobiological mechanisms that sustain these personality changes.

## 4. Neurobiological Models of Hyperreligiosity

The phenomenon of hyperreligiosity in temporal lobe epilepsy cannot be understood solely from a clinical description. Over recent decades, different neurobiological models have been proposed to explain how the human brain generates religious experiences and why they may be exacerbated in the epileptic context [8]. Among the most relevant are the limbic marker hypothesis, models based on theory of mind [18] and brain networks, and, more recently, consideration of the uncinate fasciculus as an integrative structure.

### 4.1. Limbic Marker Hypothesis

Saver and Rabin [8], in their influential article “The Neural Substrates of Religious Experience”, proposed that the limbic system functions as an emotional “marker” that tags certain experiences with transcendent significance.

This model stems from the clinical observation that many temporal epileptic seizures—particularly those with ecstatic symptoms—involve limbic structures such as the amygdala, hippocampus, and cingulate cortex. According to the authors, aberrant activation of these circuits produces a disproportionate emotional “tagging,” whereby neutral experiences acquire the value of ultimate reality, cosmic harmony, or divine contact.

The fundamental features of the religious experience in this model are as follows:

- Sense of supreme reality: the patient perceives the episode as more “real” than everyday life.
- Unity and harmony: integration of internal and external stimuli into a totalizing experience.
- Ecstasy and serenity: intense emotions of peace, joy, or fullness.
- Ineffability: difficulty or impossibility of verbally expressing the experience.

The limbic system, as the generator of emotional memory, would be responsible for the intensity and permanence of these experiences in the patient’s life history. The model aligns with what has been described as “ecstatic epilepsy” and helps explain why ictal or postictal experiences may provoke long-lasting religious conversions.

### 4.2. Theory of Mind and Brain Networks

More recently, cognitive neuroscience has approached religious experiences from the framework of the theory of mind (ToM) [18] and the dynamics of functional brain networks.

#### 4.2.1. Theory of Mind (ToM)

ToM refers to the human capacity to attribute mental states (beliefs, desires, intentions) to oneself and others. It is essential for empathy, morality, and social life. Menon [18] and other authors have linked ToM with the construction of religious beliefs: attributing intentions to invisible agents, interpreting events as messages, and generating coherent systems of belief.

#### 4.2.2. Triple Network Model (TNM)

The network model proposed by Menon and expanded by McNamara & Grafman [19] integrates three major systems:

- Default Mode Network (DMN): involves the medial prefrontal cortex, posterior cingulate cortex, and precuneus. It is activated during introspection, autobiographical memory, and self-referential thought. In religion, it contributes to spiritual reflection and the sense of continuity of the self.
- Salience Network (SN): includes the anterior insula and dorsal anterior cingulate cortex. It detects relevant internal and external stimuli, granting them emotional priority. In hyperreligiosity, it may attribute transcendent value to neutral perceptions.
- Central Executive Network (CEN): formed by the dorsolateral prefrontal cortex and posterior parietal regions. It is responsible for cognitive control and critical evaluation. Its dysfunction in pathological contexts may favor the uncritical acceptance of mystical experiences.

#### 4.2.3. Clinical and Experimental Evidence

- In bipolar disorder, manic episodes are associated with hyperactivation of the DMN, which may generate grandiose religious ideas [20].
- In schizophrenia, ToM and SN dysfunction contribute to the genesis of delusions with religious content [18].
- In studies with psilocybin and other psychedelics, disruption of DMN connectivity correlates with experiences of ego dissolution and mystical visions [21].
- Transcranial stimulation of the medial prefrontal cortex has been shown to reduce the intensity of religious beliefs [22], while stimulation of the anterior insula can induce sensations of transcendent connection [9,23].

This network model explains how mystical phenomena may arise from the interaction—or disruption—of brain systems that usually regulate meaning attribution, emotional evaluation, and critical thinking.

### 4.3. The Uncinate Fasciculus as a Possible Integrative Pathway

The uncinate fasciculus is a white matter tract described by Reil in 1809. It connects the orbitofrontal and frontopolar cortex with anterior temporal and limbic structures such as the amygdala and hippocampus [24,25,26,27,28,29,30,31,32].

Its integrative function is twofold:

1. Bidirectionality: allows emotional stimuli from the limbic system to be modulated by frontal regions.
2. Valuation and meaning: facilitates perceptual experiences, acquiring affective and moral connotation.

#### 4.3.1. Anatomical and Functional Evidence

Beyond its anatomical description, the uncinate fasciculus is increasingly recognized as a key pathway for emotion–cognition integration. By linking anterior temporal structures—including the temporal pole, amygdala, and anterior hippocampus—with the orbitofrontal and frontopolar cortices, it provides a structural substrate through which emotionally laden memories and percepts can influence higher-order decision-making, valuation, and social judgment [24,27,30,32]. Diffusion-based tractography studies have shown that microstructural indices of uncinate fasciculus integrity correlate with performance on tasks that require the integration of affective cues with cognitive demands, such as socioemotional evaluation and flexible behavioral planning [24,25,27,32].

The uncinate fasciculus has also been implicated in episodic and autobiographical memory, particularly in the retrieval of personally relevant, emotionally colored events, as well as in aspects of novelty and salience processing. Its position at the interface between limbic regions and orbitofrontal cortex allows novel or unexpected stimuli to be rapidly assigned an affective value and incorporated into the individual’s narrative and semantic frameworks [24,26,28,29,32]. In this sense, the tract acts as a conduit through which temporal-limbic “markers” of relevance can modulate frontal representations of goals, norms, and meaning, fitting well with models that highlight the role of limbic tagging in religious and ecstatic experiences [8].

#### 4.3.2. Clinical Studies

Clinically, diffusion tensor imaging studies have reported that reduced fractional anisotropy or abnormal diffusivity within the uncinate fasciculus is associated with psychotic symptoms, delusional ideation, and deficits in theory of mind and social cognition, particularly in schizophrenia and bipolar disorder [20,31]. These findings suggest that when uncinate integrity is compromised, the transmission of socioemotional and mnemonic information from the temporal lobes to frontal evaluative regions becomes less reliable, favoring misattribution of salience and the formation of rigid, idiosyncratic belief systems. In early-course schizophrenia, for example, uncinate abnormalities have been linked to both cognitive impairment and positive symptoms [31], underscoring the tract’s relevance for the construction and maintenance of coherent representations of reality.

In epilepsy, although the literature remains more limited, uncinate fasciculus alterations may likewise contribute to the propagation of temporal discharges toward orbitofrontal and frontopolar cortices, thereby shaping the phenomenology of ictal experiences. Within this framework, the uncinate fasciculus does not act in isolation, but as part of a broader frontotemporal and limbic–paralimbic network that supports valuation, autobiographical memory, and the attribution of meaning [20,24,30,31,32].

#### 4.3.3. Relation to Hyperreligiosity

Given its function as a bridge between the temporal lobes and the orbitofrontal cortex, the uncinate fasciculus could be the anatomical substrate that materializes the limbic marker hypothesis within the TNM framework. Temporal lobe epilepsy, by excessively activating this circuit, would produce exaggerated emotional tagging of neutral experiences, which would then be perceived as transcendent or divine.

Although evidence remains preliminary, studies such as the clinical case presented in this article reinforce the hypothesis that structural alterations of the uncinate fasciculus may be associated with hyperreligiosity phenomena.

Given this converging evidence, it is plausible that structural compromise of the uncinate fasciculus, as observed in our patient, could bias emotion–cognition integration and autobiographical meaning-making toward exaggerated or transcendent interpretations of otherwise neutral experiences, thereby contributing to the emergence of hyperreligiosity in the context of temporal lobe epilepsy [8,18,19,20,24,31,32].

## 5. Illustrative Clinical Case

### 5.1. Background

We present the case of a 53-year-old woman, previously healthy, with no relevant medical history and no prior psychiatric or neurological background. The patient identified herself as an atheist, with no religious practices or interest in spirituality.

### 5.2. Clinical History

The patient presented with a six-month history of holocranial headache, accompanied by daily episodes (5–6 times/day) of déjà vu sensation, described as brief and stereotyped.

**Figure 1:**
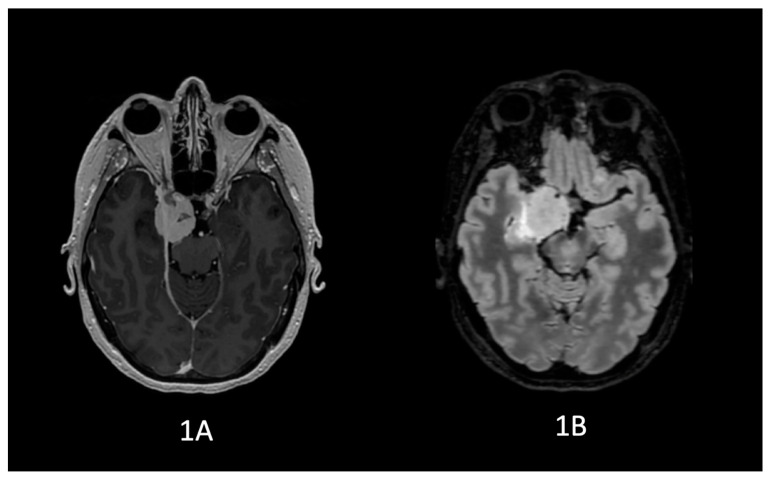

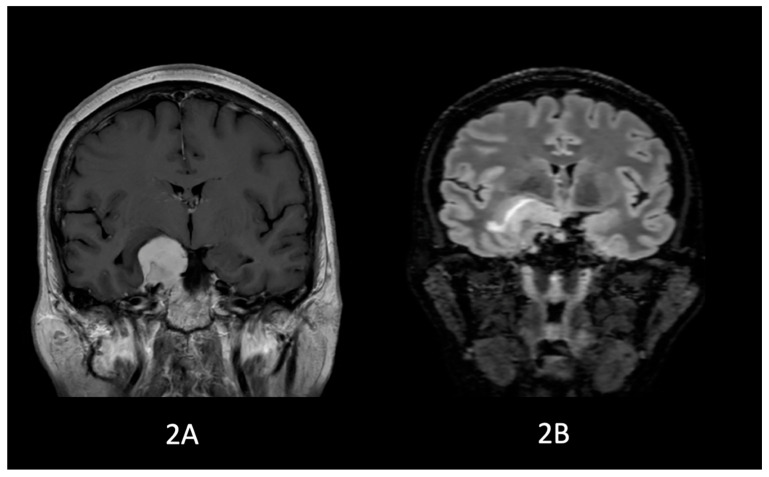
MRI study prior to surgical resection of the lesion: (**1A**,**1B**) display axial MRI images using T1-weighted sequence with gadolinium contrast and T2-weighted sequence, respectively. Note the associated edema surrounding the meningioma located in the right medial temporal lobe. Similarly, (**2A**,**2B**) present the same findings in a coronal view.

Brain MRI revealed an extra-axial lesion compatible with right cavernous sinus meningioma, in contact with the medial wall of the temporal lobe and with significant vasogenic edema in the adjacent parenchyma (Figure 1).

Treatment with levetiracetam 1000 mg/12 h was initiated, with a partial reduction in seizures. However, in subsequent weeks, the patient began to present with different episodes, characterized by a sudden, intense, and absolute conviction of the existence of God, accompanied by emotions of security and transcendent revelation.

These episodes were paroxysmal (1–2 times/day), lasted several minutes, and were accompanied by specific behaviors: spontaneous prayer, purchase of a rosary, and daily religious practice. Notably, this occurred in a previously atheist patient with no history of faith or religiosity.

Over time, although the seizures resolved spontaneously, her spiritual orientation transformed in a stable manner, shifting from an atheist position to a sustained religious conviction.

### 5.3. Complementary Studies

#### 5.3.1. Video-EEG

**Figure 2:**
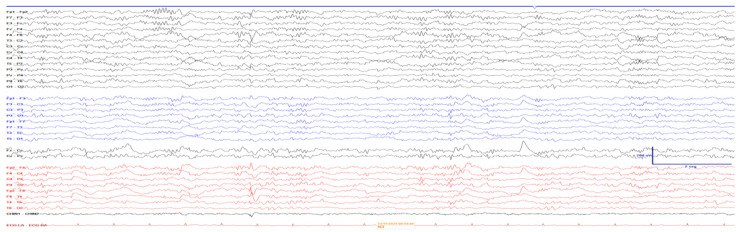
Pre-Surgical EEG. This figure presents the results of a 24 h video EEG performed on our patient. Clear evidence of irritative activity (moderately frequent bursts of sharp waves superimposed on a slowing EEG) is observed in the right temporal lobe during wakefulness and sleeping time, correlating with the clinical epileptic seizures documented.

A 24 h video-EEG recording was performed, which did not capture clinical seizures but showed irritative activity in the right temporal lobe (Figure 2). This finding supported the diagnosis of temporal lobe epilepsy (TLE) secondary to the compressive effect of the meningioma and perilesional edema.

#### 5.3.2. Diffusion Tensor Imaging (DTI) Tractography

**Figure 3:**
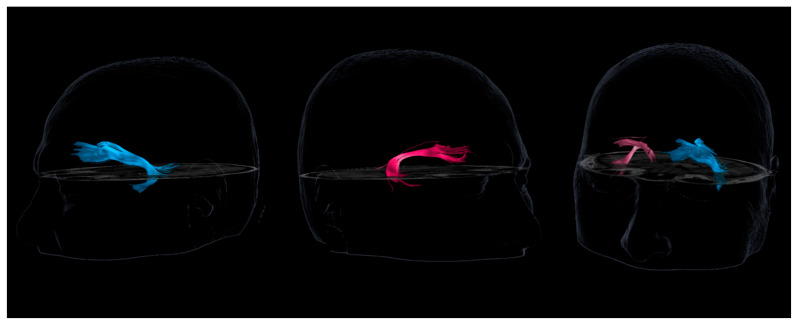
Different views of the tractography of both uncinate fascicles. This figure presents various views of the tractography of both uncinate fascicles. The left uncinate fasciculus (UF) is shown in blue, while the right one is depicted in pink. A significant difference in thickness and fiber density between the two is clearly observable.

Prior to surgery, diffusion tensor imaging tractography was performed. The study revealed displacement and thinning of the right uncinate fasciculus compared to the left (Figure 3). This finding suggested a structural alteration of the tract due to the mass effect of the tumor and edema, offering an anatomical correlate to the patient’s hyperreligiosity phenomena.

In addition to the qualitative visualization, a quantitative comparison was performed using our institutional Brainlab^®^ DTI analysis platform. This system provides automated metrics for each reconstructed tract. The right uncinate fasciculus showed a reduced streamline count (32% fewer streamlines compared to the left), decreased tract volume (28% reduction), and lower mean fractional anisotropy (FA) values (0.37 on the right vs. 0.42 on the left), consistent with displacement and partial compression of the tract by the adjacent meningioma. These measurements corroborate the visual impression of thinning and support the presence of a structural alteration rather than a purely subjective assessment. All analyses were performed using identical tracking parameters for both hemispheres to ensure reproducibility.

### 5.4. Surgical Intervention

The patient underwent right pterional craniotomy with intradural transsylvian approach, with the aim of resecting the meningioma and decompressing the temporal lobe.

The resection included the cranial and lateral portions of the tumor. However, the portion intimately adherent to the cavernous sinus was preserved due to the risk of severe neurological deficit from involvement of the internal carotid artery and cranial nerves (III, IV, VI, V1, and V2).

Continuous intraoperative neurophysiological monitoring was performed throughout the surgery, allowing preservation of neurovascular structures.

### 5.5. Postoperative Course

After surgery, the patient no longer presented paroxysmal episodes of religious conviction or déjà vu seizures. She continued on levetiracetam at the same dosage (1000 mg/12 h).

**Figure 4:**
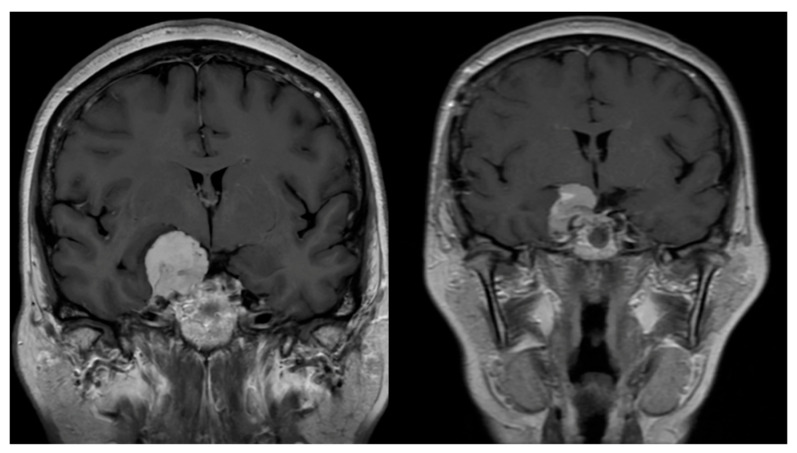
MRI study prior to surgical resection of the lesion (**left**), coronal MRI images using T1-weighted sequence with gadolinium contrast, and MRI study after surgical resection of the lesion (**right**), coronal MRI images using T1-weighted sequence with gadolinium contrast, where a decompression of the right temporal lobe can be observed.

Postoperative MRI showed adequate decompression of the right temporal lobe (Figure 4).

Interestingly, although ictal episodes of hyperreligiosity disappeared, the patient retained a stable religious orientation, praying daily and maintaining her newly acquired faith. However, she no longer experienced the ecstatic and overwhelming certainty of divine presence, but rather a more rationalized and sustained belief.

This suggests the following:

1. The paroxysmal episodes of divine conviction were directly related to epileptic activity and alteration of the uncinate fasciculus.
2. The patient’s spiritual transformation persisted beyond the cessation of seizures, reflecting how intense ictal experiences can induce lasting changes in the belief system.

### 5.6. Case Commentary

This clinical case represents, to the best of our review, one of the first to document through tractography a structural alteration of the uncinate fasciculus associated with hyperreligiosity phenomena.

- At the ictal level, the patient presented with brief episodes of absolute certainty of the existence of God.
- At the interictal level, she developed sustained religiosity that persisted even after surgery.
- The correlation with alteration of the right uncinate fasciculus supports the hypothesis that this white matter tract may act as a key modulator in the propagation of epileptic activity toward frontal regions involved in meaning attribution.

Although alterations in the uncinate fasciculus have previously been associated with hyperreligiosity, delusional ideation, and disturbances in meaning attribution—particularly in schizophrenia, bipolar disorder, and idiopathic temporal lobe epilepsy [18,24,31]—the present case adds a distinct perspective. Unlike prior studies that describe microstructural abnormalities identified through DTI in primary neuropsychiatric disorders, our patient exhibited a clearly identifiable mass effect caused by a cavernous sinus meningioma, resulting in displacement and thinning of the right uncinate fasciculus. This provides a direct and anatomically observable correlate for the patient’s religious experiences. Rather than proposing a novel mechanism, our findings extend the existing literature by illustrating how a structural, compressive lesion can modulate temporofrontal connectivity in a manner consistent with models of aberrant emotional tagging and meaning attribution.

Thus, the contribution of this case lies not in documenting the uncinate fasciculus’ involvement per se—which has been previously suggested—but in demonstrating how focal mass effect on this tract may produce a similar phenomenology to that described in idiopathic epilepsy or psychiatric disorders. This reinforces the role of frontotemporal pathways in the emergence of hyperreligiosity and supports the integrative frameworks proposed by recent network-based models.

## 6. Discussion

The association between epilepsy and religious experiences has been recognized since antiquity and has even influenced the lives of historical and religious figures. Accounts such as those of Saint Paul, with visions described as raptures to the “third heaven,” or the mystical ecstasies of Saint Teresa of Ávila, have been reinterpreted in the neurological literature as possible manifestations of temporal lobe epilepsy (TLE), with phenomena compatible with psychic auras or ecstatic epilepsy [2,3]. These historical precedents already suggested what modern research has confirmed: that there is a privileged relationship between temporal epilepsy and religious experiences.

The phenomenology of these experiences has been systematized into three main forms: ictal, postictal, and interictal [4,5,6]. Ictal experiences, although brief, may have enormous emotional intensity and include visions, voices, or the sensation of a divine presence, especially in patients with right temporal foci [4]. Postictal experiences often last for hours or days, presenting in the form of religious delusions or convictions of a transcendent mission, sometimes within the framework of postictal psychosis [4]. Finally, interictal religiosity manifests as a stable personality change, characterized by persistent hyperreligiosity, hypergraphia, moralizing discourse, and decreased sexual interest, constituting what Waxman and Geschwind described as an interictal behavioral syndrome [5,6]. These three clinical levels are not mutually exclusive and, in some patients, may coexist at different moments of the disease.

Regarding underlying mechanisms, several models attempt to explain how the brain generates religious experiences. Saver and Rabin proposed the limbic marker hypothesis, according to which the limbic system assigns a special emotional tagging to certain stimuli, generating the experience of contact with an ultimate reality and explaining the ineffability of such experiences [8]. More recently, models based on the theory of mind and the triple network model [18,19] have been developed, involving the interaction of three major brain networks: the default mode network, responsible for introspection and self-referential thought; the salience network, which detects and prioritizes emotionally charged stimuli; and the central executive network, which regulates attention and cognitive control. Alterations in the dynamics between these networks may explain why neutral stimuli acquire transcendent value in TLE, and why similar phenomena also appear in psychiatric disorders or under the influence of psychedelics [9,20,21,22,23,33].

Our clinical case fits within this framework. The patient presented paroxysmal episodes of absolute religious conviction, in which she described an unshakable certainty of the existence of God—experiences that align with the ictal phenomenology previously described [4]. An additional conceptual nuance concerns the patient’s persistent religious orientation after surgery. A more accurate interpretation is that the patient experienced a postictal or post-epileptic personality transformation, in which highly emotionally charged ictal and peri-ictal experiences underwent long-term consolidation and were integrated into her stable belief system. Similar enduring behavioral changes have been described within the classical interictal syndrome of temporal lobe epilepsy [5,6], and may be facilitated by the intense limbic emotional tagging proposed by Saver and Rabin [8]. In this framework, the patient’s lasting spiritual orientation does not reflect ongoing epileptic activity, but rather the durable incorporation of previously ictal experiences into autobiographical memory and personal identity. Most strikingly, these phenomena developed in a previously atheist woman, underscoring the transformative power of such experiences on the belief system. EEG showed irritative activity in the right temporal lobe, reinforcing the relationship between this localization and religious phenomena.

An additional finding was the identification, through tractography, of an alteration in the right uncinate fasciculus. Although this observation should not be interpreted as direct causality, it aligns with the hypothesis that temporofrontal connections play a modulatory role in meaning attribution and in the integration of emotions and cognitions [24,25,26,27,28,29,30,31,32]. In this sense, our case constitutes an illustrative contribution, but it should be understood within a broader framework in which multiple circuits intervene, rather than a single anatomical substrate.

The functional interpretation of this case can be grounded entirely on previously described models and evidence. The uncinate fasciculus has been consistently implicated in socioemotional integration, valuation processes, and frontotemporal communication [24,27,30,32]. Alterations of this tract have also been linked to disturbances in belief formation, delusional ideation, and theory of mind in schizophrenia and bipolar disorder [20,31], supporting its role as a key conduit through which limbic salience signals reach frontal evaluative regions. From the perspective of large-scale networks, our findings align with the triple network model [18] and recent accounts relating brain networks to religious cognition [19]. Disruption of temporofrontal connectivity may impair the salience network’s ability to regulate and contextualize amygdala-driven emotional tagging, consistent with mechanisms proposed both in ecstatic epilepsy and in insular/salience disturbances [8,9,23]. Thus, without invoking new mechanisms, our case extends existing models by demonstrating how a structurally visible mass effect on the uncinate fasciculus can perturb the same networks previously implicated in hyperreligiosity and meaning attribution.

Additional support for our interpretation comes from recent findings linking right-hemispheric temporal activity with increased propensity for spiritual or transcendental experiences in patients with epilepsy. In a notable diffusion and functional imaging study, Lee et al. [34] reported that individuals with higher levels of intrinsic spirituality exhibited stronger right-hemisphere lateralization, particularly involving frontotemporal and limbic structures. Their work suggests that the right temporal lobe may play a privileged role in mediating the affective, experiential, and intuitive dimensions of religiosity—dimensions that are prominently activated during ictal and peri-ictal states in temporal lobe epilepsy. Importantly, Lee and colleagues proposed that right-lateralized networks may facilitate the integration of emotional salience, autobiographical meaning, and novelty processing, aligning closely with models of limbic emotional tagging and frontotemporal valuation discussed in our review.

These findings resonate with the broader conceptual framework proposed by Devinsky and Lai [4], who emphasized that alterations in religious experience—whether ictal, postictal, or interictal—frequently stem from disturbances within temporo-limbic circuits, with a notable predominance in right-sided foci. When viewed alongside our case, this literature supports the hypothesis that the patient’s hyperreligious experiences arose not only from local irritative activity and structural compromise of the uncinate fasciculus, but also from a possible hemisphere-specific predisposition toward attributing transcendent meaning to internal stimuli. The convergence of structural alteration, right temporal epileptic activity, and established right-hemispheric involvement in spirituality strengthens the interpretive plausibility of our findings and enriches the neurobiological context within which this case should be understood.

Overall, the literature review and case analysis confirm that hyperreligiosity in temporal epilepsy is a well-described clinical phenomenon, with diverse expressions and explanations that combine phenomenological, neuroanatomical, and network perspectives. The available evidence points to the limbic system as the core of the experience, to distributed brain networks as modulators of meaning attribution, and to structural connectivity as an additional factor that may condition the way these experiences are expressed. No single theory alone explains the complexity of the phenomenon, but all provide complementary pieces to understand how brain activity can give rise to religious experiences that patients perceive as genuine transcendent revelations.

It is also important to consider potential confounding factors beyond the uncinate fasciculus itself. The patient’s cavernous sinus meningioma exerted a clear mass effect on the medial temporal lobe, accompanied by substantial vasogenic edema. This pattern of compression could have affected not only the uncinate tract but also adjacent limbic and paralimbic regions—including the amygdala, hippocampal formation, orbitofrontal cortex, and insular areas—which have been repeatedly implicated in ecstatic phenomena and altered salience attribution [8,9,23]. Thus, the patient’s hyperreligious episodes may reflect the combined impact of network-level disruption rather than an isolated tract-specific effect.

Additionally, although levetiracetam is not typically associated with hyperreligiosity, antiseizure medications can modulate mood, cognition, and behavioral activation [35,36,37]. For completeness, we acknowledge that the pharmacological treatment may have contributed modestly to the patient’s overall neurocognitive state, even if it is unlikely to account for the distinct paroxysmal religious experiences described [35]. Taken together, these factors highlight the complexity of interpreting single-case neurobehavioral phenomena and underscore the need for cautious, network-based explanations.

## 7. Conclusions and Limitations

Hyperreligiosity in temporal lobe epilepsy is a complex phenomenon that may manifest at different stages of the disease and adopt highly diverse forms. The case presented illustrates how these experiences may arise in a defined clinical context and how, even after seizure control, they may leave a lasting imprint on the patients’ belief system.

These findings highlight the importance of considering the religious and spiritual dimension in the evaluation of patients with temporal epilepsy—not only as a clinical curiosity, but as an integral part of their life experience. Likewise, they emphasize the need for further research into the neurobiological mechanisms underlying these experiences, with special attention to the interaction between brain networks, limbic structures, and personal and cultural factors.

The main limitations of this work include its nature as a single case, which prevents generalization of the findings, and the inherent constraints of the neuroimaging techniques employed. Moreover, religious experience exceeds any purely neurological explanation and requires integration of cultural, biographical, and social perspectives.

In short, the study of hyperreligiosity associated with temporal lobe epilepsy offers a privileged window into the relationship between brain and spirituality—a field that still poses more questions than answers and demands a rigorous multidisciplinary approach.

## Data Availability

No new data were created or analyzed in this study. Data sharing is not applicable to this article.
